# Invisible and undernourished: food insecurity among international graduate students in the USA as an overlooked nutrition equity challenge

**DOI:** 10.1017/S1368980026103292

**Published:** 2026-07-24

**Authors:** Abu Ansar Md Rizwan

**Affiliations:** Nutritional Sciences, Texas Tech University, USA

**Keywords:** Food insecurity, International students, Graduate students, Dietary acculturation, Nutrition equity, Cardiometabolic risk, Cultural food access, Higher education

## Abstract

Food insecurity among international graduate students in the USA is a nutrition equity challenge that remains largely unaddressed by the nutrition science community. Prevalence estimates range from 22 % to 46 % across institutions, substantially exceeding the national household average, driven by converging financial constraints, restricted employment opportunities and limited access to culturally appropriate foods. These conditions promote rapid dietary acculturation towards energy-dense, low-nutrient food patterns with well-established associations with insulin resistance, hypertension and cardiometabolic risk. Despite a growing empirical base, longitudinal evidence on dietary quality and health outcomes in this population is virtually absent, and university nutrition support systems are poorly adapted to the cultural dietary needs of an internationally diverse student body. This commentary calls on nutrition researchers, dietitians and universities to recognise food insecurity among international graduate students as a substantive public health nutrition concern warranting targeted research and culturally responsive institutional action.

The USA hosts more than one million international students annually, a figure that grew by 7 % in 2023 alone^(1)^. These students, predominantly graduate level and predominantly from Asia, contribute over USD 43·8 billion to the US economy and support nearly 400 000 jobs each academic year^(1)^. They are recruited aggressively, celebrated publicly and yet remain largely overlooked when it comes to their nutritional health and food security. A growing body of evidence now makes clear that food insecurity among international graduate students in the USA is far more prevalent than commonly recognised, and that the nutrition science community has yet to meaningfully engage with either the problem or its health consequences.

Prevalence estimates of food insecurity among international college students in the USA range from 22 % to 46 %, depending on institution and measurement approach^(2–4)^, substantially exceeding the 13·7 % national household food insecurity rate reported by the USDA in 2024^(5)^. At a large public university in the southwestern USA, 61 % of surveyed international students reported low or very low food security^(4)^. At a North Texas university, 85 % of international graduate student participants reported limited access to nutritious and culturally appropriate foods, with 70 % reporting hunger due to financial constraints^(6)^. A study of graduate students at a major private university found that 17 % experienced food insecurity during the academic year, with racial and ethnic minority status, a category that disproportionately includes international students, as a significant independent predictor^(7)^. These prevalence estimates are corroborated by direct comparisons with domestic peers. In a stratified analysis of 4819 undergraduate and graduate students at a large public university, international student status independently increased the odds of food insecurity (*v*. food security) among undergraduates and of marginal (*v*. high) food security among graduate students, relative to domestic students^(8)^. These figures represent a systematic pattern with direct implications for dietary quality, cardiometabolic health and academic functioning that the nutrition community has been slow to address.

Several converging factors place international graduate students at elevated nutritional risk. Graduate stipends, where they exist at all, frequently fall below living wage thresholds, particularly in university towns where housing and food costs have risen sharply^(6,9)^. Many international students arrive with limited personal savings, limited familiarity with local food systems and limited cooking experience outside their home dietary traditions. Employment opportunities during the academic year are constrained by programme requirements and visa conditions that limit working hours, leaving students with narrow economic margins and limited flexibility to improve their food access situation independently^(9)^. This financial vulnerability is compounded by a categorical lack of access to public assistance. Unlike domestic students, international students are ineligible for federal need-based financial aid, including grants, work-study and federal loans^(10)^, and for the Supplemental Nutrition Assistance Program (SNAP), the principal food assistance safety net available to food-insecure US citizens and qualifying residents^(11)^. Graduate stipends, where available, therefore represent students’ only financial buffer against food insecurity, with no public fallback if that buffer fails.

Cultural and dietary dimensions add a further layer of vulnerability that standard food insecurity assessments frequently fail to capture. The concept of nutrition security, which incorporates not only caloric adequacy but also access to nutritionally adequate and culturally appropriate food, is particularly relevant here^(12)^. International students from South Asia, East Asia and sub-Saharan Africa report significant difficulty accessing culturally familiar foods both at campus food pantries and through local grocery infrastructure^(4,6)^. This matters nutritionally: dietary acculturation under conditions of economic constraint drives rapid transitions towards cheap, energy-dense, low-nutrient foods, a pattern associated with increased cardiometabolic risk^(13)^. The long-term dietary and health consequences of sustained food insecurity during graduate training remain poorly characterised in this population, but established mechanistic pathways are concerning. Chronic food insecurity is associated with poor diet quality, insulin resistance, hypertension, dyslipidaemia and elevated cardiovascular risk through neuroendocrine stress responses, altered eating patterns and sustained nutritional inadequacy^(14,15)^.

Campus food pantries represent a genuine institutional effort to address student food insecurity, but evidence consistently shows that international students underutilise them. Studies identify stigma, inconvenient hours, language barriers, and, critically, the absence of culturally appropriate food options as primary deterrents^(16)^. A student whose dietary staples are rice, lentils or fermented foods receives limited nutrition security from a pantry stocked with canned beans and instant pasta. This cultural mismatch between available food assistance and actual dietary needs is itself a nutrition equity problem that warrants specific institutional attention.

Nutrition science and public health communities have a clear role to play that has not yet been adequately taken up. Three priorities stand out. First, researchers should systematically include international student populations in studies of food insecurity and dietary quality, with explicit attention to cultural food access, dietary acculturation and cardiometabolic outcomes. Most existing work is single-institution, cross-sectional and limited to food security status; longitudinal evidence on dietary quality and health trajectories is almost entirely absent^(4,6,7)^. Second, registered dietitians and nutrition educators embedded in university health systems should develop culturally adapted dietary counselling resources for international students navigating food access constraints. Generic dietary advice calibrated to Western dietary patterns is of limited practical value to a student from South Asia or East Africa trying to eat well on a constrained budget in a mid-sized American university town. Third, universities recruiting internationally should evaluate whether their food support infrastructure, pantries, meal programmes and emergency food funds are designed to serve the actual population using them, including their cultural diversity. Designing food assistance around the needs of a predominantly domestic student body and then offering it unchanged to an internationally diverse graduate population is equality, not equity.

More than one million international students are studying in the USA. A substantial proportion is food insecure. A meaningful proportion of those are experiencing poor diet quality and the associated health risks that the nutrition science community understands well in every other context it studies^(14,15)^. The gap between what is known about food insecurity and cardiometabolic health, and what is being done to understand and address it in this specific population, is one that nutrition researchers, dietitians and university health systems are well positioned to close. It is time to start.
